# Potentially inappropriate medication use as predictors of hospitalization for residents in nursing home

**DOI:** 10.1186/s12877-023-04165-w

**Published:** 2023-08-02

**Authors:** Hyun-Woo Chae, Jing Zhao, Young-Mi Ah, Kyung Hee Choi, Ju-Yeun Lee

**Affiliations:** 1 College of Pharmacy and Research Institute of Pharmaceutical Sciences, Seoul National University, 1 Gwanak-ro, Gwanak-gu, Seoul, 08826 Republic of Korea; 2 College of Pharmacy, Yeungnam University, 280 Daehak-Ro, Gyeongsan, Gyeongsangbuk, 38541 Republic of Korea; 3 College of Pharmacy, Gachon University, 191 Hambakmoero, Yeonsu-gu, Incheon, 21936 Republic of Korea

**Keywords:** Potentially inappropriate medication, Nursing home, Hospitalization, Emergency department

## Abstract

**Background:**

Hospitalization of nursing home (NH) residents impose a significant healthcare burden. However, there is still a lack of information regarding the risk of hospitalization from inappropriate prescribing in NH residents. We aimed to estimate the nationwide prevalence of potentially inappropriate medication (PIM) use among NH residents using the Korean tool and 2019 Beers criteria and to assess their associations with hospitalization or emergency department (ED) visits.

**Methods:**

We included older adults aged 65 years or above who were admitted to NHs between July 2008 and December 2018 using national senior cohort database. The prevalence of PIM use based on the Korean medication review tool and Beers criteria on the date of admission to NH was estimated. And the adjusted hazard ratios (aHRs) of polypharmacy, numbers of PIM, each PIM category for hospitalization/ED visits within 30 days of admission to NH was calculated using Cox proportional hazard model to show the association.

**Results:**

Among 20,306 NH residents, the average number of medications per person was 7.5 ± 4.7. A total of 89.3% and 67.9% of the NH residents had at least one PIM based on the Korean tool and 2019 Beers criteria, respectively. The risk of ED visits or hospitalization significantly increased with the number of PIMs based on the Korean tool (1–3: aHR = 1.24, CI 1.03–1.49; ≥4: aHR = 1.46, CI 1.20–1.79). Having four or more PIMs based on the Beers criteria increased the risk significantly (aHR = 1.30, CI 1.06–1.53) while using 1–3 PIMs was not significantly associated (aHR = 1.07, CI 0.97–1.19). Residents with any potential medication omission according to the Korean criteria, were at 23% higher risk of hospitalization or ED visits (aHR = 1.23, CI 1.07–1.40).

**Conclusions:**

This study demonstrated that PIMs, based on the Korean tool and Beers criteria, were prevalent among older adults living in NHs and the use of PIMs were associated with hospitalization or ED visits. The number of PIMs based on the Korean tool showed dose-response increase in the risk of hospitalization or ED visits.

**Supplementary Information:**

The online version contains supplementary material available at 10.1186/s12877-023-04165-w.

## Introduction

The global population is getting older because of its increased life expectancy. In 2015, the proportion of older adults aged 65 years or above reached 8.5%, and it is expected to be 16.7% by 2050 [1]. South Korea’s population is currently experiencing one of the world’s most rapid aging phenomena. According to a report by the United Nations, the proportion of older adults aged 65 years or above in South Korea is projected to increase by 23% points between 2019 and 2050 [2]. In response to this aging trend, the National Health Insurance Service (NHIS) in South Korea implemented a long-term care (LTC) insurance scheme in July 2008. As a result, there has been a steady increase in the number of LTC benefit recipients and LTC institutions [3, 4].

Many residents in nursing home (NH) have one or more chronic diseases, which expose them to polypharmacy [5]. Previous research showed that almost 40% of residents in NHs take more than five medications, and up to 19.4% of residents take more than seven medications [6]. According to a systematic review by Bronskill et al., the average number of medications taken by residents in NH ranged from 3.8 to 16.6 [7]. Due to the high medication usage in this population, there is an increased likelihood of taking potentially inappropriate medication (PIM), which can lead to drug-related problems or harm. Krustev et al. studied the association between polypharmacy or PIMs and drug-related problems and found that older adults who were administered polypharmacy (i.e., five or more medications) have twice the risk of taking PIMs, and about three out of ten patients taking PIMs can experience drug-related problems [8].

Residents in NHs tend to get hospitalized or visit the emergency department (ED) frequently. According to US data, more than one-fourth of long-stay NH residents get hospitalized each year, and many of these cases could potentially have been avoided [9, 10]. Such hospitalizations or ED visits of NH residents are considered a burden both to the residents themselves and healthcare system. In addition, previous researches have indicated that medication use, including PIMs, is associated with hospitalization in older adults [11]. In the earlier study based on longitudinal data, inappropriate prescribing as per the Beers criteria in NH residents was found to be associated with subsequent hospitalization and death [12]. Another study conducted in a skilled nursing facility suggested that the use of psychotropics might be associated with an increased rate and risk of all hospitalizations [13]. However, there are still lack of information regarding the risk of hospitalization from the use of PIMs in nursing home residents.

To minimize the potential harm related to medication use in vulnerable older adults, several screening tools for older adults’ prescriptions have been developed, including the Beers criteria of the American Geriatrics Society, STOPP/START criteria from Europe, and other adapted tools for specific countries [14, 15]. Moreover, there are tools developed for older adults, particularly those residing in nursing facilities, such as STOPP/START criteria for the US nursing home setting and Norwegian General Practice-Nursing Home criteria [16, 17]. Recently, a medication review tool for residents in Korean long-term care facilities (Korean MR tool-LTCF) has been developed, aimed at detecting PIM use [18]. It is an explicit criteria tool based on disease categories, consisting of 77 items categorized into five types: PIM in general (General-PIM), PIM due to drug interactions (DDI-PIM), PIM under specific diseases or conditions (Ds-PIM), PIM needed for monitoring and dose adjustment (Dose-PIM), and potential medication omissions (Omission-PIM). This tool was developed through a literature review and two rounds of a modified Delphi survey with expert panel representation.

Even though a pilot study has been conducted in two Korean NHs to test the applicability of the Korean MR tool-LTCF [19], there is still a lack of studies using Korean MR tool-LTCF in detecting PIMs and predicting ED visits or hospital admission in Korean NH residents. Therefore, we aimed to investigate the nationwide prevalence of PIMs and their association with hospitalization or ED visits in NH residents using the Korean MR tool-LTCF and the 2019 Beers criteria.

## Methods

### Data source

For this study, we used a senior cohort database from the National Health Insurance Service (NHIS)-Senior Cohort (NHIS-SC). The NHIS provides health insurance to all citizens in South Korea as the single national insurer and also covers LTC insurance [3]. Older adults with LTC grade are eligible for beneficiaries of LTC insurance. The LTC grade is determined based on the diagnosis of dementia and LTC approval score, which is derived from assessing physical and cognitive abilities [3].

This senior cohort database consists of 18 years (2002–2019) of cumulative records of 511,953 stratified randomly sampled older adults aged 60 to 80 years, as of 2008, with an annual replacement of 8% of adults who turned 60 from 2009 to 2018; all the data is de-identified. The data contains demographic information, medical resource utilization data, and LTC service utilization data, including the grade for LTC services of the population.

### Study population

In this cross-sectional study, we analyzed the medication use at the time of NH admission. Therefore, we included older adults aged 65 years or above who were admitted to NHs between July 2008 and December 2018, according to the time of enforcement of LTC insurance by NHIS. We excluded residents who did not have any medication on the day of admission to NHs or residents who had less than 30 consecutive days of stay from the admission day. Furthermore, if a resident was readmitted to the NH within seven days of discharge, it was considered a single episode.

### Prevalence of PIM use and polypharmacy

We estimated the prevalence of PIM use with the medications used on the date of admission to NH for each resident. For each resident, PIMs were identified by the Korean MR tool-LTCF (hereafter, Korean criteria) and Beers criteria, which is the most utilized and recognized screening tool in South Korea. We could not include two items of Dose-PIM (> 200 mg/day of oral iron supplement, start dose of tramadol), one item of DDI-PIM (when to take bisphosphonate), and three items of Omission-PIM (annual influenza vaccination, pneumococcal vaccination at least once, oral supplemental nutrition for malnourished patients with dysphagia/chewing disorder) from the Korean criteria due to the limited information in claims database. Similarly, we did not include the following: items of PIMs related to kidney function, eight items of General-PIMs, and one item from the Ds-PIM of Beers criteria. The included items by the modified PIM definitions in the analysis and the list of Anatomical Therapeutic Chemical codes for each item are listed in Supplementary Tables 1, 2. When measuring the number of medications, we excluded topical agents, such as ointment, cream, eyedrop, and eardrop, except inhalers. In case of fixe dose combination products, individual ingredients were counted. We defined polypharmacy and excessive polypharmacy as five or more and ten or more concurrent medication use, respectively [20].

### Association of PIM use with hospitalization or ED visit

The outcomes of interest were the first hospitalization or ED visits within 30 days of admission to NH. Due to the limitations of claims data, we were unable to distinguish between unplanned and planned hospitalizations. Therefore, for the purpose of this study, we included any hospitalization regardless of whether it was planned or unplanned. ED visits with a length of stay of one day were classified differently from hospitalizations, which were defined as stays of two or more days, including those through the ED. To assess the association between PIM use, which include both the overall number of PIMs and specific categories of PIMs, and hospitalization/ED visits, we employed a multivariable-adjusted Cox proportional hazard analysis. Furthermore, to gain a more comprehensive understanding of the specific effects of PIMs on ED visits or hospitalization among NH elderly residents, we also examined the associations of individual PIM items.

### Statistical analysis

The demographic characteristics and prevalence of PIM use in the study population were described using descriptive statistics. A t-test and a chi-square test were used to compare continuous and categorical variables, respectively, between residents who had experienced ED visit/hospitalization. To assess the risk of hospitalization or ED visits associated with PIM use, including the total number of PIMs, specific categories of PIMs, and individual PIM items, we employed the multivariable-adjusted Cox proportional hazard model. We adjusted several potential confounding variables, including age, sex, insurance type, disability level, LTC grade, Charlson comorbidity index (CCI), and underlying diseases such as dementia, hypertension, diabetes, ischemic heart disease, heart failure, chronic obstructive pulmonary disease, asthma, peptic ulcer disease, history of fractures, history of pneumonia, and cancer (Supplementary Table 3). Demographic were measured on the day of admission to nursing home. And preexisting diagnoses were identified for the 1 year before admission to nursing home using International Classification of Diseases (ICD) codes. aHRs of each component of PIMs were analyzed by adjusted multivariate Cox proportional hazard as described above. All statistical analyses were conducted using SAS Enterprise Guide software, version 7.1 (SAS Institute, Inc., Cary, NC) with a 95% CI, and a *p*-value < 0.05 was considered statistically significant.

## Results

A total of 20,306 older adults with one or more medications were admitted to NHs between July 2008 and December 2018 (Table 1). The mean age of the study population was 78.7 ± 5.6 years, and 70.3% of the residents were female. The common comorbidities among residents were dementia, hypertension, and diabetes, with a prevalence of 76.3%, 75.2%, and 44.5%, respectively. Of the 20,306 residents, 1,877 (9.2%) residents experienced hospitalization (6.5%) or ED visit (2.7%) within one month of admission to the NH.

**Table 1:** Baseline characteristics of the study population (N = 20,306)

Characteristics	N (%)
**Sex**	
Male	6,038 (29.7)
Female	14,268 (70.3)
**Age (years), mean ± SD**	78.7 ± 5.6
65–69	1,503 (7.4)
70–74	3,120 (15.4)
75–79	5,877 (28.9)
80–84	6,522 (32.1)
≥ 85	3,284 (16.2)
**Insurance type**	
Health insurance	16,789 (82.7)
Medical aid	3,517 (17.3)
**Disability level**	
Non-disabled	13,056 (64.3)
Moderate disability	4,004 (19.7)
Severe disability	3,246 (16.0)
**LTC grade**	
Grade 1	2,165 (10.7)
Grade 2	5,119 (25.2)
Grade 3 and below	13,022 (64.1)
**CCI, mean ± SD**	3.7 ± 2.2
0	493 (2.4)
1 ~ 4	13,480 (66.4)
≥ 5	6,333 (31.2)
**Comorbidities**	
Dementia	15,492 (76.3)
Hypertension	15,267 (75.2)
Diabetes	9,044 (44.5)
COPD	5,684 (28.0)
PUD	5,634 (27.8)
History of fractures	5,086 (25.1)
IHD	4,093 (20.2)
History of pneumonia	3,132 (15.4)
Heart failure	3,122 (15.4)
Angina	3,078 (15.2)
Asthma	2,933 (14.4)
Parkinson’s disease	2,839 (14.0)
Cancer	1,388 (6.8)

LTC, Long-Term Care; CCI, Charlson Comorbidity Index; COPD, Chronic Obstructive Pulmonary Disease; PUD, Peptic Ulcer Disease; IHD, Ischemic Heart Disease

The average number of medications was 7.5 ± 4.7, and around 70.5% of patients were taking polypharmacy. Furthermore, 29.9% of patients were exposed to excessive polypharmacy. Among the study population, 67.7% and 89.3% were identified as taking one or more of PIMs based on the Beers and Korean criteria, respectively. The largest proportion of residents was exposed to General-PIMs (62.8%) and Omission-PIMs (77.2%) based on the Beers and Korean criteria, respectively (Table 2).

**Table 2:** Prevalence of PIM use in the residents on the day of admission to nursing home

Variables	Total (N = 20,306) N (%)	With hospitalization/ ED visits (N = 1,877) N (%)	Without hospitalization/ ED visits (N = 18,429) N (%)	*p*-value
**No. of medications**, mean ± SD	7.5 ± 4.7	8.2 ± 5.2	7.4 ± 4.7	< 0.001
1 ~ 4	5,990 (29.5)	492 (26.2)	5,498 (29.8)	< 0.001
5 ~ 9	8,241 (40.6)	697 (37.1)	7,544 (40.9)	
≥ 10	6,075 (29.9)	688 (36.7)	5,387 (29.2)	
**Total PIMs**				
** Beers criteria**, mean ± SD	2.8 ± 2.0	2.9 ± 2.1	2.8 ± 2.0	0.001
0	6,513 (32.1)	545 (29.0)	5,968 (32.4)	0.002
1 ~ 3	12,829 (63.2)	1,210 (64.5)	11,619 (63.0)	
≥ 4	964 (4.7)	122 (6.5)	842 (4.6)	
** Korean criteria**, mean ± SD	3.9 ± 2.7	4.1 ± 2.8	3.9 ± 2.6	< 0.001
0	2,171 (10.7)	136 (7.3)	2,035 (11.0)	< 0.001
1 ~ 3	10,744 (52.9)	924 (49.2)	9,820 (53.3)	
≥ 4	7,391 (36.4)	817 (43.5)	6,574 (35.7)	
**General-PIMs**				
** Beers criteria**, mean ± SD	1.9 ± 1.2	2.0 ± 1.2	1.9 ± 1.2	0.000
0	7,550 (37.2)	635 (33.8)	6,915 (37.5)	0.002
≥ 1	12,756 (62.8)	1,242 (66.2)	11,514 (62.5)	
** Korean criteria**, mean ± SD	1.8 ± 1.2	1.8 ± 1.2	1.8 ± 1.2	0.10
0	9,597 (47.3)	858 (45.7)	8,739 (47.4)	0.16
≥ 1	10,709 (52.7)	1,019 (54.3)	9,690 (52.6)	
**Ds-PIMs**				
** Beers criteria**, mean ± SD	2.1 ± 1.3	2.1 ± 1.3	2.1 ± 1.3	0.19
0	10,314 (50.8)	918 (48.9)	9,396 (51.0)	0.09
≥ 1	9,992 (49.2)	959 (51.1)	9,033 (49.0)	
** Korean criteria**, mean ± SD	2.0 ± 1.4	2.0 ± 1.5	2.0 ± 1.4	< 0.001
0	9,367 (46.1)	776 (41.3)	8,591 (46.6)	< 0.001
≥ 1	10,939 (53.9)	1,101(58.7)	9,838 (53.4)	
**DDI-PIMs**				
** Beers criteria**, mean ± SD	1.1 ± 0.5	1.2 ± 0.5	1.1 ± 0.5	0.09
0	15,557 (76.6)	1,415 (75.4)	14,142 (76.7)	0.19
≥ 1	4,749 (23.4)	462 (24.6)	4,287 (23.3)	
** Korean criteria**, mean ± SD	1.7 ± 1.3	1.9 ± 1.4	1.7 ± 1.3	0.006
0	16,462 (81.1)	1,488 (79.3)	14,974 (81.3)	0.037
≥ 1	3,844 (18.9)	389 (20.7)	3,455 (18.7)	
**Omission-PIMs**				
** Korean criteria**, mean ± SD	2.1 ± 1.1	2.2 ± 1.2	2.1 ± 1.1	< 0.001
0	4,632 (22.8)	318 (16.9)	4,314 (23.4)	< 0.001
≥ 1	15,674 (77.2)	1,559 (83.1)	14,115 (76.6)	

PIM, Potentially Inappropriate Medication; Ds-PIM, PIM under specific diseases or conditions; DDI-PIM, PIM due to drug interactions

Of the 1,877 residents who were hospitalized or visited ED within a month of their admission, 36.7% had excessive polypharmacy compared to 29.2% of residents who did not require hospitalization or an ED visit. Among the residents who were hospitalized or visited ED, the average numbers of PIMs based on the Beers and Korean criteria were 2.9 ± 2.1 and 4.1 ± 2.8, respectively, which was significantly higher than those who did not require hospitalization or an ED visit (2.8 ± 2.0, p = 0.001 and 3.9 ± 2.6, p < 0.001, respectively, of PIMs, based on respective criteria).

Cox-proportional hazard model analysis showed that the risk of hospitalization or ED visits within a month after admission to NH increased with the use of PIMs after adjusting for confounding factors (Table 3). Residents who used four or more of PIMs based on the Beers and Korean criteria at the time of NH admission had a 30% (adjusted hazard ratio (aHR) 1.30, 95% CI 1.06–1.59) and 46% (aHR, 1.46, 95% CI 1.20–1.79) higher risk of ED visit or hospitalization, respectively, than those who did not use PIMs. Moreover, residents who used one to three PIMs based on the Korean criteria had a 51% higher risk of visiting the ED (aHR 1.51, 95% CI 1.06–2.14) than those without PIM based on Korean criteria, whereas those who used four or more PIMs based on the Korean criteria had almost 80% higher risk of ED visits (aHR 1.79, 95% CI 1.23–2.60).

**Table 3:** Association between medication use and PIM use with risk of ED visits or hospitalization (N = 20,306)

Variables	ED visits or hospitalization aHR (95% CI)	ED visits aHR (95% CI)	Hospitalization aHR (95% CI)
**Number of medications**			
1–4	1	1	1
5–9	0.98 (0.87–1.10)	1.03 (0.83–1.27)	0.96 (0.84–1.11)
≥ 10	1.20 (1.06–1.36) ^*^	1.22 (0.97–1.54)	1.19 (1.03–1.38)^†^
**Total PIMs**			
** Beers criteria**			
0	1	1	1
1–3	1.07 (0.97–1.19)	1.14 (0.94–1.38)	1.05 (0.93–1.18)
≥ 4	1.30 (1.06–1.59) ^*^	1.50 (1.03–2.19)^†^	1.22 (0.96–1.56)
** Korean criteria**			
0	1	1	1
1–3	1.24 (1.03–1.49)^†^	1.51 (1.06–2.14)^†^	1.14 (0.92–1.42)
≥ 4	1.46 (1.20–1.79) ^*^	1.79 (1.23–2.60) ^*^	1.35 (1.06–1.70) ^*^
**General-PIMs**			
** Beers criteria**			
0	1	1	1
≥ 1	1.11 (1.00-1.22)^†^	1.13 (0.95–1.35)	1.10 (0.98–1.23)
** Korean criteria**			
0	1	1	1
≥ 1	1.03 (0.94–1.13)	1.04 (0.88–1.23)	1.02 (0.92–1.14)
**Ds-PIMs**			
** Beers criteria**			
0	1	1	1
≥ 1	1.10 (1.00-1.22)^†^	1.17 (0.97–1.40)	1.08 (0.96–1.21)
** Korean criteria**			
0	1	1	1
≥ 1	1.17 (1.06–1.29) ^*^	1.27 (1.06–1.51) ^*^	1.13 (1.01–1.27)^†^
**DDI-PIMs**			
** Beers criteria**			
0	1	1	1
≥ 1	1.04 (0.93–1.16)	1.17 (0.96–1.42)	0.99 (0.87–1.12)
** Korean criteria**			
0	1	1	1
≥ 1	1.10 (0.98–1.23)	1.11 (0.90–1.37)	1.10 (0.96–1.25)
**Omission-PIMs**			
** Korean criteria**			
0	1	1	1
≥ 1	1.23 (1.07–1.40) ^*^	1.17 (0.92–1.49)	1.25 (1.06–1.48) ^*^

ED, Emergency Department; Ds-PIM, PIM under specific diseases or conditions; DDI-PIM, PIM due to drug interactions; Adjusted variables were age, sex, Long-Term Care grade, Charlson Comorbidity Index, disease (dementia, hypertension, diabetes, Chronic Obstructive Pulmonary Disease, Peptic Ulcer Disease, history of fractures, Ischemic Heart Disease, heart failure, asthma, history of pneumonia, cancer) ^*^p < 0.01,†p < 0.05

Residents with one or more Ds-PIMs (Beers aHR 1.10, 95% CI 1.00-1.22; Korean aHR 1.17, 95% CI 1.06–1.29) or General-PIMs based on Beers criteria (aHR 1.11, 95% CI 1.00-1.22) had a significantly higher risk of ED visits or hospitalization. Residents with any Omission-PIM, the classification type according to the Korean criteria, were at 23% higher risk of hospitalization/ED visits (aHR 1.23, 95% CI 1.07–1.40).

The most prevalent item among the General-PIM that may affect hospitalization/ED visits in NH residents was the “use of proton pump inhibitor (PPI),” with 8.3%, which increased the risk of hospitalization/ED visits by 22% (aHR 1.22, 95% CI 1.05–1.42). The “use of ketorolac” as a General-PIM by Beers criteria has been associated with a four-fold increased risk of hospitalization or ED visits (aHR 3.95, 95% CI 1.27–12.28). Regarding Ds-PIM, only one item from the Korean criteria, “diuretics use in patients with urinary incontinence,” showed a significant association with an aHR of 1.38 (95% CI 1.00-1.90). Concurrent use of warfarin + macrolides/quinolones and oral anticoagulants + antiplatelet drugs significantly increased the risk of hospitalization or ED visits, with an aHR of 3.24 (95% CI 1.04–10.12) and 1.75 (95% CI 1.25–2.44), respectively. Regarding Omission-PIM, “omission of vitamin D and calcium supplements in patients with a history of fall or risk of osteoporosis” and “omission of vitamin D in patients with severe renal impairment” increased the risk of hospitalization or ED visits with an aHR of 1.13 (95% CI 1.00-1.27) and 1.27 (95% CI 1.01–1.60), respectively (Table 4 and Supplementary Table 4).

**Table 4:** Significant items of PIMs that increase the risk of visiting ED or hospitalization in nursing home residents (N = 20,306)

Items		Frequency N (%)	ED visits or hospitalization aHR (95% CI)
**General-PIM**			
Ketorolac^**‡**^		8 (0.0)	3.95 (1.27–12.28)^†^
Dronedarone/Amiodarone^**‡**^		61 (0.3)	2.05 (1.18–3.54) ^*^
Insulin (short- or rapid-acting)^**‡**^		1,233 (6.1)	1.34 (1.14–1.58) ^*^
PPI^**||**^		1,679 (8.3)	1.22 (1.05–1.42) ^*^
**Ds-PIM**			
**Disease or Syndrome**	**Drug**		
Urinary incontinence	Diuretics^**§**^	296 (1.5)	1.38 (1.00-1.90)^†^
**DDI-PIM**			
**Object Drug**	**Interacting Drug**		
Warfarin	Macrolides (excluding azithromycin) Quinolones^**§**^	8 (0.0)	3.24 (1.04–10.12)^†^
Oral anticoagulants	Antiplatelet drugs^**§**^	194 (1.0)	1.75 (1.25–2.44) ^*^
**Omission-PIM**			
**Disease or Syndrome**	**Omission Drugs**		
Severe renal impairment	Vit D supplements^**§**^	588 (2.9)	1.27 (1.01–1.60)^†^
Experiences a fall, or is at high risk of osteoporosis	Vit D and calcium supplements^**§**^	8,528 (42.0)	1.13 (1.00-1.27)^†^

ED, Emergency Department; Ds-PIM, PIM under specific diseases or conditions; DDI-PIM, PIM due to drug interactions; PPI, Proton Pump Inhibitor; Vit D, Vitamin D

Adjusted variables were age, sex, Long-Term Care grade, Charlson Comorbidity Index, disease (dementia, hypertension, diabetes, Chronic Obstructive Pulmonary Disease, Peptic Ulcer Disease, history of fractures, Ischemic Heart Disease, heart failure, asthma, history of pneumonia, cancer)

^*^p < 0.01,†p < 0.05

^**‡**^Included in Beers criteria only ^**§**^Included in Korean criteria only ^**||**^Included in both Beers criteria and Korean criteria

## Discussion

In this cross-sectional study, we estimated the nationwide prevalence of PIM use based on the Beers and Korean criteria and assessed their association with the increased risk of hospitalization or ED visits in older adults living in NHs. We found that more than two-thirds of the NH residents are exposed to PIMs regardless of the type of criteria used, which is consistent with previous studies [21–23]. One study analyzing claims data with a sample of 8,835 NH residents found that 81.6% of residents were exposed to PIMs according to the 2019 Beers criteria during the first year after NH admission [21]. Similarly, a research team in Belgium reported that among 1,410 residents in 54 NHs participating in a cluster-controlled trials, 88.3% of them were prescribed PIMs based on the Beers and STOPP/START criteria [22]. The prevalence of PIM use varies depending on the study population, method of measurement, and most importantly, criteria used to define PIM. The prevalence of PIM use based on the newly developed Korean criteria, which is specific to the residents of NHs, was significantly higher than that based on the Beers criteria. The largest difference between these two criteria is the inclusion of Omission-PIM, which can detect omitted medications that need to be used for one’s condition or disease but have not been prescribed. About 77.2% of residents had at least one medication that needs to be prescribed according to the Korean criteria. The number of total PIMs identified based on the Korean criteria was larger than that based on the Beers criteria, possibly owing to the absence of Omission-PIM in the Beers criteria. This tendency has been observed in other studies as well. Boland et al. identified PIMs using both the Beers and STOPP/START criteria through expert review of medication regimen among twenty patients admitted to geriatric ward of a teaching hospital and showed that the STOPP/START criteria, which includes criteria for Omission-PIM, detected up to 122 more items than the 2012 Beers criteria [23].

Of the 20,306 NH residents, approximately 10% of residents visited the ED or were hospitalized during the first month of the NH stay. These frequencies were similar to those observed in previous studies. A study in Australia reported that 18.0% of residents had unplanned hospitalization within 90 days of admittance to a nursing home [24]. Moreover, a study conducted in Canada found that nearly a quarter of the newly admitted residents to long-term care facilities visited the ED at least once in a year after the admission [25].

After adjusting for confounding factors, we showed that the use of PIMs in NH residents is associated with an increased risk of hospitalization or ED visits. The risk of adverse outcomes increased with the number of PIMs, particularly when using the Korean criteria for identification. Those exposed to four or more PIMs had an aHR of 1.46 (95% CI 1.20–1.79), and those exposed to one to three PIMs had an aHR of 1.24 (95% CI 1.03–1.49) compared to those without PIMs. These results confirmed the previous findings. One study conducted in the US showed that NH residents with PIMs based on the Beers criteria were at a 28% higher risk of hospitalization [12]. Price et al. also reported that PIM use in NH residents was significantly associated with hospitalizations, with an adjusted odds ratio of 1.21 (95% CI 1.10–1.34) [26].

Analysis of the association of categories or individual items of PIMs with adverse outcomes revealed that ketorolac and dronedarone/amiodarone in General-PIM were highly associated with ED visits or hospitalization. Short/rapid-acting insulin and PPI also increased the risk of adverse outcomes and were relatively common in the residents. Similarly, Lohman et al. investigated the hospitalization risk in Medicare home health nursing patients and reported that patients taking hormone and gastrointestinal medications had 17% and 33% greater risk of hospitalization than those not taking PIMs in these respective classes [27]. Regarding DDI-PIM, residents taking oral anticoagulants + antiplatelets had a 75% higher risk of ED visits or hospitalization in our study. Few studies have assessed the associations between DDI-PIM and hospitalization in NH residents. However, a previous study reported the risk of hospitalization were increased in community-dwelling older adults exposed to DDI-PIM, including concurrent use of antiplatelets and NSAIDs without PPI, multiple antiplatelets without PPI, antiplatelets plus oral anticoagulants without PPI, and ACE inhibitors/ARB with NSAID [28].

In our study, more than 40% of residents were exposed to one of the Omission-PIMs, the absence of vitamin D and calcium supplements in patients with risk of fall or osteoporosis, which increased the risk of ED visits or hospitalization by 13%. One study conducted in Spain demonstrated similar results, detecting Omission-PIMs using START criteria in 81 older adults living in a NH [29]. The most prevalent Omission-PIM was calcium and vitamin D supplementation in patients with known osteoporosis, which accounted for 15% of the residents.

To the best of our knowledge, this is the first study that applies the Korean criteria to investigate the prevalence of PIMs and their association with a negative outcome in NH residents using claims data. Moreover, as we used nationwide claims data from NHIS, which covers the entire Korean population with health insurance and medical aid, we consider our study population representative. Additionally, we analyzed all the individual items of respective criteria to find not only the prevalence in NH residents but also its association with ED visits or hospitalization.

However, there are several limitations to this study. First, we analyzed the medications that older adults were taking on the day of admission to NHs. There may be differences between these medications and those taken continuously after admission to NHs. However, older adults tend to maintain their prescriptions without changes after admission to NHs. It is also possible that medication used solely on the day of admission to the NH are included in our analysis. Nevertheless, it is important to consider that most older adults are transferred to NHs when they are in a stable condition. Therefore, we believe that analyzing only the medications taken on the day of admission will not have a significant impact on the results. Second, owing to limited information in the claims data, we could not identify and analyze certain PIMs, such as Dose-PIM, DDI-PIM related to bisphosphonate administration, and PIMs related to renal function. Furthermore, the claims data used in the study did not allow for distinguishing between hospitalizations that were unplanned or planned. Therefore, there is a possibility of overestimation in the association between PIM use and hospitalization. To address these limitations, additional information, such as laboratory data or patient interviews, would be needed.

Third, we could not confirm whether residents were taking medications as prescribed in the claims data. Moreover, over-the-counter medications were not considered. Fourth, it is important to note that we did not analyze the specific reasons for hospitalization and ED visits in this study. Therefore, we could not confirm whether the observed hospitalization/ED visits were directly related to medication/PIM use. It is possible that there were other factors unrelated to PIM use that contributed to these outcomes. However, in older adults, polypharmacy and the presence of PIMs are known to be factors that contribute adverse health outcomes such as falls, or adverse drug reactions, which can subsequently lead to ED visits and hospitalization [30–32].

Our study findings demonstrated that the use of PIM is prevalent among older adults in NHs, and such use is associated with an increased risk of ED visits or hospitalization. Unlike other countries such as Canada or Australia [33, 34], Korea does not currently have established systems in place for conducting medication reviews specially for older adults admitted to long-term care facilities. Therefore, it is suggested that medication reviews at the time of admission to NH are crucial in order to identify inappropriate prescriptions and reduce the risk of hospitalization or ED visits. Furthermore, considering that our study findings indicate the relationship between the number of PIMs identified by the Korean criteria with the occurrence of ED visits or hospitalization, it can be inferred that the Korean criteria, developed specifically for NH residents in Korea, are suitable for monitoring PIM use among older adults residing in NHs in Korea.

## Conclusions

Our study findings underscore the widespread occurrence of PIMs among older adults residing in NHs, as assessed by both the Korean tool and Beers criteria. Importantly, the results demonstrate a clear association between the use of PIMs and an elevated risk of hospitalization or ED visits. Notably, the analysis reveals a dose-response relationship, wherein an increasing number of PIMs identified using the Korean tool is linked to a higher likelihood of hospitalization or ED visits.

## Electronic supplementary material

Below is the link to the electronic supplementary material.

Supplementary Material 1

## Data Availability

The datasets used in the study can be accessed from the National Health Institute service, but their use is limited due to licensing and not intended for public release. However, data will be shared on reasonable request to the corresponding author with the permission of the National Health Institute service.

## Abbreviations

CCI: Charlson comorbidity index
DDI-PIM: PIM due to drug interactions
Dose-PIM: PIM needed for monitoring and dose adjustment
Ds-PIM: PIM under specific diseases or conditions
ED: Emergency department
LTC: Long-term care
LTCF: Long-term care facilities
MR: Medication review
NH: Nursing home
NHIS: National Health Insurance Service
Omission-PIM: Potential medication omissions
PIM: Potentially inappropriate medication
