# Supplementary material for: Potentially inappropriate medication use as predictors of hospitalization for residents in nursing home
Source: BMC Geriatr. 2023 Aug 2;23:467. doi: 10.1186/s12877-023-04165-w (PMC10394923; doi:10.1186/s12877-023-04165-w)
Supplement: Supplementary file 1 — Supplementary Material 1 [file 12877_2023_4165_MOESM1_ESM.pdf]

Supplementary material

Title: Potentially inappropriate medication uses as predictors of hospitalization for residents in nursing home

- Table S1. Definitions for PIM exposure adapted from the 2019 Beers Criteria
- Table S2. Definitions for PIM exposure adapted from the Korean criteria
- Table S3. Diagnostic code of chronic diseases/conditions included in the study
- Table S4. Prevalence and association of individual PIM items with the risk of ED visits and hospitalization

Table S1. Definitions for PIM exposure adapted from the 2019 Beers Criteria

Type	Criteria	ATC code
**Anticholinergic drugs**		
General-PIM	First-generation antihistamines	R06AA, R06AB, R06AD, R06AX, N05BB
General-PIM	Anticholinergic muscle relaxant	M03BA, M03BC, M03BX
General-PIM	Anticholinergic antispasmodics	A03AA, A03AB, A03BA, A03BB, A03CA
General-PIM	Antiparkinsonian agents	M03BC, N04AA, N04AC
Ds-PIM	Strong anticholinergic agents except antimuscarinics in patients with benign prostatic hyperplasia and urinary retention	A03AA, A03AB, A03BA, A03BB, A03CA, G04BD, G04BX, M03BA, M03BC, M03BX, N04AA, N04AC, N05AA, N05AB, N05AE, N05AF, N05AG, N05AH, N05AX, N05BB, N06AA, N06AB, R06AA, R06AB, R06AD, R06AE, R06AX
Ds-PIM	Strong anticholinergic drugs in patients with dementia or cognitive decline	
DDI-PIM	Two or more strong anticholinergic drugs	
**NSAIDs**		
General-PIM	Oral NSAIDs (including over-the-counter drugs) use in high-risk patients (high-risk patient group: patients over 75 years of age, patients taking oral steroids, anticoagulants, and antithrombotic drugs)	M01AB, M01AC, M01AE, M01AG, M01AX
Ds-PIM	NSAIDs in patients with renal failure	
Ds-PIM	Nonselective NSAIDs in peptic ulcer patients	
DDI-PIM	Combination of warfarin and NSAIDs	
DDI-PIM	Combination of oral NSAIDs (including over-the-counter drugs) and oral steroids	
Ds-PIM	Continued use of NSAIDs/ Coxib (including over-the-counter drugs) in patients with heart failure	M01AB, M01AC, M01AE, M01AG, M01AH, M01AX
**Cardiovascular drugs**		
Ds-PIM	Alpha-blockers as first-line treatment for hypertension in patients without BPH	C02CA, C04AX, G04CA
Ds-PIM	Alpha-blockers with urinary incontinence (all types) in women	
DDI-PIM	Combination of loop diuretics and peripheral α-1 blocker	
Ds-PIM	Nonselective peripheral alpha-1 blockers in patients with syncope	
General-PIM	Dronedarone/amiodarone	C01BD01, C01BD07
Ds-PIM	Dronedarone in patients with heart failure	C01BD07
DDI-PIM	Combination of warfarin and amiodarone	C01BD01
General-PIM	Digoxin for first-line treatment of atrial fibrillation or of heart failure	C01AA
General-PIM	Nifedipine, immediate release	C08CA

Ds-PIM	Nondihydropyridine CCBs in patients with heart failure	C08DA, C08DB
DDI-PIM	Combination of ACEI/ARB and diuretics	C03AA, C03BA, C03CA C03DA, C03DB
DDI-PIM	Combination of loop diuretics and lithium	C03CA
DDI-PIM	Combination of loop diuretics and peripheral α-1 blocker	
DDI-PIM	Combination of ACEI and ARB	C09AA, C09CA, C09DX
DDI-PIM	Combination of ACEI/ARB and diuretics	
DDI-PIM	Combination of ACEI and lithium	C09AA
**Endocrine drugs**		
General-PIM	Estrogen with or without progestins	C03CA, C03CX
Ds-PIM	Estrogen in women with urinary incontinence (all types)	
General-PIM	Insulin (short/rapid-acting)	A10AB, A10AD
General-PIM	Megestrol	L02AB01
General-PIM	Sulfonylureas, long-acting	A10BB01, A10BB12
Ds-PIM	Thiazolidinediones (pioglitazone, rosiglitazone) in patients with heart failure	A10BG03, A10BG04
**Gastrointestinal drugs**		
General-PIM	Metoclopramide	A03FA01
Ds-PIM	Metoclopramide in patients with Parkinson's disease	
General-PIM	Use of full therapeutic doses PPI for >8 weeks: increased risk of C. difficile infection, bone loss, and fracture risk\	A02BC
**Antiplatelet & anticoagulants**		
General-PIM	Oral NSAIDs (including over-the-counter drugs) use in high-risk patients (high-risk patient group: patients over 75 years of age, patients taking oral steroids, anticoagulants, and antithrombotic drugs)	B01AA, B01AB, B01AC, B01AE, B01AF, B01AX
Ds-PIM	Cilostazol in patients with heart failure	B01AC23
DDI-PIM	Combination of warfarin and amiodarone	B01AA03
DDI-PIM	Combination of warfarin and ciprofloxacin	
DDI-PIM	Combination of warfarin and macrolides (excluding azithromycin)	
DDI-PIM	Combination of warfarin and trimethoprim-sulfamethoxazole	
DDI-PIM	Combination of warfarin and NSAIDs	
**CNS drugs**		
General-PIM	Nonbenzodiazepine, benzodiazepine receptor agonist hypnotics (ie, "Z-drugs")	N05CF
Ds-PIM	Nonbenzodiazepine, benzodiazepine receptor agonist hypnotics with dementia or cognitive impairment	
General-PIM	Antidepressants	N06AA, N06AB
Ds-PIM	Tertiary TCAs with syncope	N06AA
General-PIM	Antipsychotics	N05A, N05AA, N05AB,

Ds-PIM	Antipsychotics (except quetiapine, clozapine, pimavanserin) with Parkinson's disease	N05AD, N05AE, N05AF, N05AG, N05AH, N05AL, N05AX
Ds-PIM	Antipsychotics with dementia or cognitive impairment	
Ds-PIM	Antipsychotics with syncope	N05AA, N05AH
General-PIM	Barbiturates	N01AF, N03CA, N05CA,
General-PIM	Benzodiazepines	N03AE, N05BA, N05BA, N05CD
Ds-PIM	Benzodiazepines with dementia or cognitive impairment	
DDI-PIM	Combination of benzodiazepines and opioid analgesics	
DDI-PIM	Combination of opioids and gabapentin, pregabalin	N03AX
DDI-PIM	Combination of phenytoin and trimethoprim-sulfamethoxazole	N03AB02
DDI-PIM	Use of opioids, benzodiazepines, antidepressants, antipsychotics, sedatives, and antiepileptics in patients with fall history	N03AA, N03AB, N03AE, N03AF, N03AG, N03AX, N05A, N05AA, N05AB, N05AD, N05AE, N05AF, N05AG, N05AH, N05AL, N05AN, N05AX, N05B, N05BA, N05BE, N05CD, N05CF, N06AA, N06AB, N06AG, N06AX
**Opioid**		
General-PIM	Meperidine	A03FA01
General-PIM	Ketorolac	M01AB15
DDI-PIM	Combination of benzodiazepines and opioid analgesics	N01AH, N02AA, N02AB, N02AD, N02AE, N02AF, N02AX, R05DA
DDI-PIM	Combination of gabapentin/ pregabalin and opioid analgesics	
**Others**		
General-PIM	Antithrombotics	B01AC
General-PIM	Desmopressin	H01BA
General-PIM	Ergoloid mesylates	C04AE01, C04AA01
Ds-PIM	Acetylcholine esterase inhibitor use in patients experiencing syncope and bradycardia	N06DA
DDI-PIM	Combination of warfarin and sulfamethoxazole/trimethoprim	J01EE01
DDI-PIM	Combination of phenytoin and sulfamethoxazole/trimethoprim	
DDI-PIM	Combination of warfarin with macrolide antibacterial agents (except azithromycin) or quinolones	J01FA, J01MA
DDI-PIM	Combination of ciprofloxacin and theophylline	R03DA04
DDI-PIM	Combination of theophylline and cimetidine	
DDI-PIM	Combination of NSAIDs and corticosteroids	H02AA, H02AB, H02BX

DDI-PIM	Combination of ciprofloxacin and theophylline	J01MA02
DDI-PIM	Combination of theophylline and cimetidine	A02BA01
DDI-PIM	Combination of ACEI and lithium	N05AN01
DDI-PIM	Combination of loop diuretics and lithium	

PIM, Potentially Inappropriate Medication; Ds-PIM, PIM under specific diseases or conditions; DDI-PIM, PIM due to drug interactions; NSAIDs, Non-Steroidal Anti-Inflammatory Drugs; CNS, Central Nervous System; BPH, Benign Prostatic Hyperplasia; GI, Gastrointestinal Diseases; SSRI, Selective Serotonin Reuptake Inhibitors; ACEI, Angiotensin Converting Enzyme Inhibitors; ARB, Angiotensin Receptor Blockers; COPD, Chronic Obstructive Pulmonary Disease; PPI, Proton Pump Inhibitors; CCB, Calcium Channel Blockers; Vit D, Vitamin D

Table S2. Definitions for PIM exposure adapted from the Korean criteria

Type	Criteria	ATC code
**Anticholinergic drugs**		
General-PIM	First-generation antihistamines	R06AA, R06AB, R06AD, R06AX, N05BB
General-PIM	Anticholinergic muscle relaxant	M03BA, M03BC, M03BX
General-PIM	Anticholinergic antispasmodics	A03AA, A03AB, A03BA, A03BB, A03CA
Ds-PIM	Strong anticholinergic drugs in constipation patients	A03AA, A03AB, A03BA, A03BB, A03CA, G04BD, G04BX, M03BA, M03BC, M03BX, N04AA, N04AC, N05AA, N05AB, N05AE, N05AF, N05AG, N05AH, N05AX, N05BB, N06AA, N06AB, R06AA, R06AB, R06AD, R06AE, R06AX
Ds-PIM	Strong anticholinergic agents in patients with benign prostatic hyperplasia and urinary retention	
Ds-PIM	Strong anticholinergic drugs in patients with dementia, delirium, or cognitive decline	
Ds-PIM	Strong anticholinergic agents in patients with closed-angle glaucoma	
DDI-PIM	Two or more strong anticholinergic drugs	
**NSAIDs**		
Ds-PIM	Continued use of NSAIDs/Coxib (including over-the-counter drugs) in patients using three or more antihypertensive drugs	M01AB, M01AC, M01AE, M01AG, M01AH, M01AX
DDI-PIM	Combination of diuretics (especially loop diuretics) and NSAIDs/Coxib	
Ds-PIM	NSAIDs/Coxib in patients with renal failure	
DDI-PIM	Combination of NSAIDs/Coxib and ACEI/ ARB	
DDI-PIM	Combination of NSAIDs/Coxib and SSRIs	
Ds-PIM	Continued use of NSAIDs/Coxib (including over-the-counter drugs) in patients with heart failure	
Omission-PIM	Recommend PPI when a patient using antiplatelet drugs (especially a patient using two types of antiplatelet drugs) and NSAIDs (including over-the-counter drugs) for more than one week together without an appropriate gastroprotective agent	M01AB, M01AC, M01AE, M01AG, M01AX
General-PIM	Oral NSAIDs (including over-the-counter drugs) use in high-risk patients (high-risk patient group: patients over 75 years of age, patients taking oral steroids, anticoagulants, and antithrombotic drugs)	
DDI-PIM	Combination of anticoagulants and NSAIDs	
DDI-PIM	Combination of oral NSAIDs (including over-the-counter drugs) and oral steroids	
Ds-PIM	Nonselective NSAIDs in peptic ulcer patients	
Ds-PIM	COX2-selective inhibitors in patients with cardiovascular disease	M01AH

**Antihypertension drugs**		
Ds-PIM	Continued use of NSAIDs (including over-the-counter drugs) in patients using three or more antihypertensive drugs	C02AC, C02DB, C03AA, C03BA, C03CA, C03DA, C03DB, C04AX, C08CA, C08DA, C08DB, G04CA, C07AA, C07AB, C07AG, C09AA, C09CA, C09DX
Ds-PIM	Alpha-blockers as first-line treatment for hypertension in patients without BPH	C02CA, C04AX, G04CA,
Ds-PIM	Alpha-1 blocker in patients with orthostatic hypotension	
Ds-PIM	Continuous use of loop diuretics for simple edema in patients without heart failure, liver cirrhosis, chronic renal failure, or nephrotic syndrome	C03CA
Ds-PIM	Nonselective beta-blockers (exception, sotalol) in diabetic patient	C07AA, C07AB, C07AG,
Ds-PIM	Nonselective beta-blockers in uncontrolled asthma and chronic obstructive pulmonary disease	
Ds-PIM	CCB in constipation patients	C08CA, C08DA, C08DB,
Ds-PIM	Diuretics in people with incontinence	C03AA, C03BA, C03CA
DDI-PIM	Combination of diuretics (especially loop diuretics) and NSAIDs	C03DA, C03DB
DDI-PIM	Combination of ACEI and ARB	C09AA, C09CA, C09DX
DDI-PIM	Combination of NSAIDs/Coxib and ACE inhibitor/ARB	
**PPI**		
General-PIM	Use of full therapeutic doses PPI for >8 weeks: increased risk of C. difficile infection, bone loss, and fracture risk	A02BC
Omission-PIM	Recommend PPI when a patient using antiplatelet drugs (especially a patient using two types of antiplatelet drugs) and NSAIDs (including over-the-counter drugs) for more than one week together without an appropriate gastroprotective agents	
**Antiplatelet & anticoagulants**		
General-PIM	Oral NSAIDs (including over-the-counter drugs) use in high-risk patients (high-risk patient group: patients over 75 years of age, patients taking oral steroids, anticoagulants, and antithrombotic drugs)	B01AA, B01AB, B01AC, B01AE, B01AF, B01AX
DDI-PIM	Combination of oral anticoagulants with antiplatelet drugs. Not applied when the same doctor prescribes those drugs at the same time, and the patient recognizes it.: increased risk of bleeding.	
DDI-PIM	Combination of anticoagulants and NSAIDs	B01AC, B01AX

Omission-PIM	Recommend PPI when a patient using antiplatelet drugs (especially a patient using two types of antiplatelet drugs) and NSAIDs (including over-the-counter drugs) for more than one week together without an appropriate gastroprotective agent	
Omission-PIM	Check the use of antiplatelet agents for secondary prevention of ischemic lesions in patients with past myocardial infarction, coronary stenting, coronary artery bypass surgery, cerebrovascular stent, past stroke, transient ischemic attack, and peripheral arterial vascular disease (except if anticoagulants are being used)	
Omission-PIM	Check the use of oral anticoagulants in chronic atrial fibrillation	B01AA, B01AB, B01AE, B01AF, B01AX
**Steroids**		
General-PIM	Oral NSAIDs (including over-the-counter drugs) use in high-risk patients (high-risk patient group: patients over 75 years of age, patients taking oral steroids, anticoagulants, and antithrombotic drugs)	H02AA, H02AB, H02BX
Ds-PIM	A diabetic patient who have been on oral steroids for more than one week and does not have blood glucose monitoring	
Ds-PIM	Oral steroid use instead of inhaled steroid use in patients with asthma	
DDI-PIM	Combination of oral NSAIDs (including over-the-counter drugs) and oral steroids	
Omission-PIM	Check if the patient is taking Vit D and calcium supplements in cases where a patient stays indoors only, or experiences a fall, or is at high risk of osteoporosis, or patients taking oral steroids for more than one month	
**Bisphosphonate**		
Ds-PIM	Bisphosphonate use in patients with chronic renal failure confirmed as CrCl < 30 mL/min in renal function tests	M05BA
Ds-PIM	Oral bisphosphonate prescription in patients with the active esophageal disease, dysphagia, and coma	
Omission-PIM	Recommendation of calcium/ Vit D supplementation and, if necessary, bisphosphonate in patients using oral steroids	
Omission-PIM	Check whether osteoporosis treatment is prescribed in patients diagnosed with osteoporosis (bone density T-score < 2.5) or in patients with past fragility fractures (osteoporotic fractures)	
Omission-PIM	Check if the patient is taking Vit D and calcium supplements in cases where a patient stays indoors only, or experiences a fall, or is at high	

	risk of osteoporosis, or patients taking oral steroids for more than one month	
Omission-PIM	Check if calcium/Vit D supplementation is prescribed in patients treated with bisphosphonate and denosumab	
**CNS drugs**		
General-PIM	Continued use (more than 30 days) or regular daily use of hypnotics (e.g., z-drugs, benzodiazepines)	N03AA, N03AE, N05BA, N05BA, N05BB, N05CA, N05CC, N05CD, N05CF, N06AA
General-PIM	Over-the-counter drugs for inducing sleep (e.g., doxylamine, diphenhydramine)	R06AA
General-PIM	Tricyclic antidepressants	N06AA
General-PIM	Benzodiazepines	N03AE, N05BA, N05BA, N05CD
DDI-PIM	Combination of benzodiazepines and opioid analgesics	
Ds-PIM	Use of opioids, benzodiazepines, antidepressants, antipsychotics, sedatives, and antiepileptics in patients with fall history	N03AA, N03AB, N03AE, N03AF, N03AG, N03AX, N05A, N05AA, N05AB, N05AD, N05AE, N05AF, N05AG, N05AH, N05AL, N05AN, N05AX, N05B, N05BA, N05BE, N05CD, N05CF, N06AA, N06AB, N06AG, N06AX
General-PIM	Multiple prescriptions within each class of hypnotics/sedatives, including Z-drugs, antidepressants, benzodiazepines, and antipsychotics	
Ds-PIM	Oral, nasal decongestants in insomnia patients	C01CA, R01BA, R03CA
Ds-PIM	Antipsychotics other than clozapine and quetiapine in patients with Parkinson's disease	N05A, N05AA, N05AB, N05AD, N05AE, N05AF, N05AG, N05AH, N05AL, N05AX
Ds-PIM	Multiple prescriptions within each class of hypnotics/sedatives, including Z-drugs, antidepressants, benzodiazepines, and antipsychotics	N03AA, N03AB, N03AE, N03AF, N03AG, N03AX, N05A, N05AA, N05AB, N05AD, N05AE, N05AF, N05AG, N05AH, N05AL, N05AX, N05BA, N05CD, N05CF, N06AA, N06AB, N06AG, N06AX
**Opioid**		
Ds-PIM	Use of opioids, benzodiazepines, antidepressants, antipsychotics, sedatives, and antiepileptics in patients with fall history	N01AH, N02AA, N02AB, N02AD, N02AE, N02AF, N02AX, R05DA
DDI-PIM	Combination of benzodiazepines and opioid analgesics	
Omission-PIM	Check short-acting opioid analgesics for sudden pain control in patients taking long-acting opioid analgesics to relieve cancer pain, etc.	
Omission-PIM	Check whether prophylactic laxatives are prescribed for patients taking regular opioid analgesics	
**Vitamin D and calcium supplements**		

Omission-PIM	Check the use of Vit D in patients with severe renal impairment (CrCl < 30 mL/min)	A11CC, A12AX
Omission-PIM	Check if the patient is taking Vit D and calcium supplements in cases where a patient stays indoors only, or experiences a fall, or is at high risk of osteoporosis, or patients taking oral steroids for more than one month	A02AA, A02AC, A11CC, A12AA, A12AX
Omission-PIM	Recommendation of calcium/ Vit D supplementation and, if necessary, bisphosphonate in patients using oral steroids	
Omission-PIM	Check if calcium/Vit D supplementation is prescribed in patients treated with bisphosphonate and denosumab	
**Bronchodilator inhalers**		
Ds-PIM	Oral steroid use instead of inhaled steroid use in patients with asthma	R03AC, R03AK, R03AL, R03BA, R03BB
Omission-PIM	Check regular use of long-acting bronchodilator inhalers in patients with chronic obstructive pulmonary disease	
**Others**		
General-PIM	Short-acting nifedipine	C08CA05
General-PIM	Long-acting sulfonylurea use	A10BB
General-PIM	Hormone replacement therapy with estrogen	G03CA, G03CX
General-PIM	Oral theophylline use	R03DA04
General-PIM	Metoclopramide	A10BA02
General-PIM	Oxybutynin for the treatment of overactive bladder	G04BD04
General-PIM	Metoclopramide and clebopride in Parkinson's disease patients	A03FA
General-PIM	Fluoxetine	N06AB03
Ds-PIM	Metformin in patients with end-stage renal failure or dialysis	A10BA02
Ds-PIM	Acetylcholine esterase inhibitor use in patients experiencing syncope and bradycardia	N06DA
DDI-PIM	Combination of warfarin and sulfamethoxazole/trimethoprim	J01EE01
DDI-PIM	Combination of warfarin with macrolide antibacterial agents (except azithromycin) or quinolones	J01FA, J01MA
DDI-PIM	Combination of ciprofloxacin and theophylline	R03DA04, J01MA02
DDI-PIM	Combination of phenytoin and sulfamethoxazole/trimethoprim	N03AB02
Omission-PIM	Check the statin treatment for secondary prevention of cardiovascular disease in patients with a past history of myocardial infarction, coronary stenting, coronary artery bypass surgery, cerebrovascular stent, past stroke, transient ischemic attack, and peripheral arterial disease	C10AA

Omission-PIM	Check if folic acid was prescribed for patients prescribed methotrexate for rheumatoid arthritis	162601AT, L04AX03
Omission-PIM	Check use of laxatives when constipation-causing drug use is inevitable in patients with persistent constipation	A06AB, A06AC, A06AD, A06AG

PIM, Potentially Inappropriate Medication; Ds-PIM, PIM under specific diseases or conditions; DDI-PIM, PIM due to drug interactions; NSAIDs, Non-Steroidal Anti-Inflammatory Drugs; CNS, Central Nervous System; BPH, Benign Prostatic Hyperplasia; GI, Gastrointestinal Diseases; SSRI, Selective Serotonin Reuptake Inhibitors; ACEI, Angiotensin Converting Enzyme Inhibitors; ARB, Angiotensin Receptor Blockers; COPD, Chronic Obstructive Pulmonary Disease; PPI, Proton Pump Inhibitors; CCB, Calcium Channel Blockers; Vit D, Vitamin D

Table S3. Diagnostic code of chronic diseases/conditions included in the study

Disease	Diagnostic codes (ICD-10)
Heart failure	I09.9, I11.0, I13.0, I13.2, I25.5, I42.0, I42.6-I43.8, I50-I50.9, I97.1, P29.0
Atrial fibrillation	I48-I48.9
Cardiovascular disease	G45, I63, I64, I20-I25, I21, I22, I25.2, I70-I74, I77.1, I79.0, K55.1, K55.8, K55.9, Z95
Syncope	I95, R00.1, R55
Dementia	F00~F03, F05.1, G30~G31.9
Cognitive decline	R41, R54
Parkinson's disease	G20-G23
Fall	S02, S03.0, S06-S07, S12, S13.1-S13.3, S22, S23.1, S23.2, S32, S33.1-S33.3, S42, S43.0-S43.3, S52, S53.0, S53.1, S62.0, S62.1, S72, S73.0, S82, S83.0, S83.1, S92.0, T02, T08, T10, T12, T14.2, W00-W19
Insomnia	F51, F51.0, F51.2, F51.8, F51.9, G47, G47.0, G47.2, G47.8, G47.9
End-Stage Renal Disease	N18.5, Z90.5, Z99.2
Renal failure	I12.0, I13,1, I13.2, N18, N183, N184, N18.5, N19, N25.0, Z49, Z940, Z99.2
Liver cirrhosis	I85.0, I86.4, K70.4, K71.0, K72.1-K72.9, K76.5-K76.7
Rheumatoid arthritis	M05-M06
Retention of urine	R33, R39.1
Urinary incontinence	N39.3-N39.4, R32
BPH	N40
Type 2 diabetes	E11-E14
COPD	J40-J44, J47, J60-J67.9, J68.4, J70.1, J70.3
Asthma	J45- J46
Pneumonia	J09-J18
PUD	K22, K25-K28
Esophagitis	K20-K21.0, K22-K22.4, K22.6-K23.8
Constipation	K59-K59.0
Osteoporosis	M36.0, M80.0-M81
Closed-angle glaucoma	H40.2
Cancer	C00-D48

BPH, Benign Prostatic Hyperplasia; COPD, Chronic Obstructive Pulmonary Disease; PPI, Proton Pump Inhibitors

Table S4. Prevalence and association of individual PIM items with the risk of ED visits or hospitalization

Criteria	Source‡	N (%) (N=20,306)	ED visits or Hospitalization aHR (95%CI)
**General-PIM**			
Benzodiazepines	B/K	4,512 (22.2)	1.02 (0.91-1.14)
Oral NSAIDs (including over-the-counter) use in high-risk patients (high-risk patient group: patients over 75 years of age, patients taking oral steroids, anticoagulants, and antithrombotic drugs)	B/K	2,276 (11.21)	1.07 (0.93-1.23)
Long-acting sulfonylurea	B/K	1,947 (9.6)	0.98 (0.83-1.15)
First-generation antihistamines	B/K	1,708 (8.4)	1.08 (0.92-1.26)
Proton Pump Inhibitor	B/K	1,679 (8.3)	1.22 (1.05-1.42) *
Anticholinergic antidepressant	B	1,204 (5.9)	0.91 (0.75-1.11)
Tricyclic antidepressants (except paroxetine)	K	1,015 (5.0)	0.86 (0.69-1.07)
Metoclopramide	B/K	220 (1.1)	1.30 (0.90-1.87)
Antispasmodics	B/K	204 (1.0)	1.32 (0.89-1.96)
Alpha-blockers as first-line treatment for hypertension in patients without BPH	B/K	151 (0.7)	0.92 (0.55-1.56)
Anticholinergic muscle relaxant	B/K	113 (0.6)	0.86 (0.45-1.66)
Short-acting nifedipine	B/K	34 (0.2)	0.57 (0.14-2.28)
Hormone replacement therapy with estrogen	B/K	5 (0.0)	2.69 (0.38-19.09)
Antipsychotics	B	4,539 (22.4)	1.07 (0.96-1.20)
Insulin (short- or rapid-acting)	B	1,233 (6.1)	1.34 (1.14-1.58) *
Z-drugs	B	1,361 (6.7)	0.95 (0.79-1.15)
Antiparkinsonian anticholinergic agents	B	648 (3.2)	0.95 (0.72-1.24)
Digoxin	B	561 (2.8)	1.12 (0.87-1.45)
Megestrol	B	73 (0.4)	1.15 (0.65-2.06)
Desmopressin	B	71 (0.4)	1.42 (0.76-2.65)
Dronedarone/Amiodarone	B	61 (0.3)	2.05 (1.18-3.54) *
Barbiturates	B	19 (0.1)	0.94 (0.24-3.78)
Ketorolac	B	8 (0.0)	3.95 (1.27-12.28)†
Dipyridamole, oral short-acting	B	6 (0.0)	0
Meperidine	B	2 (0.0)	3.99 (1.28-12.40) *
Ergoloid mesylate,Isoxsuprine	B	0 (0.0)	0
Hypnotics (e.g. z-drugs, benzodiazepines)	K	5,442 (26.8)	1.02 (0.92-1.13)
Multiple prescriptions within each class of hypnotics/sedatives, including z-drugs, antidepressants, benzodiazepines, and antipsychotics	K	2,280 (11.2)	0.91 (0.79-1.06)

Oral theophylline		K	261 (1.3)	1.00 (0.69-1.46)
Fluoxetine		K	161 (0.8)	0.99 (0.60-1.65)
Oxybutynin		K	90 (0.4)	1.31 (0.71-2.45)
Over-the-counter drugs for inducing sleep (e.g. doxylamine, diphenhydramine)		K	2 (0.0)	0
**Ds-PIM**				
**Disease or Syndrome**	**Drug(s)**			
Dementia	Strong anticholinergic drugs	B/K	5,769 (28.4)	1.11 (0.99-1.23)
	Antipsychotics	B	4,169 (20.5)	1.10 (0.98-1.24)
	Benzodiazepines	B	3,594 (17.7)	1.02 (0.90-1.16)
	Zolpidem, Eszopiclone	B	1,067 (5.3)	0.98 (0.79-1.21)
Constipation	Strong anticholinergic drugs	K	3,305 (16.3)	1.07 (0.95-1.21)
	Calcium channel blockers	K	3,172 (15.6)	1.05 (0.92-1.18)
Fall history	Use of opioids, benzodiazepines, antidepressants, antipsychotics, sedatives, and antiepileptics	B/K	2,952 (14.5)	1.14 (0.95-1.37)
BPH, urinary retention	Strong anticholinergic agents	K	1,374 (6.8)	1.14 (0.97-1.36)
	Strong anticholinergic agents (except antimuscarinics)	B	1,138 (5.6)	1.19 (0.99-1.42)
Urinary incontinence	Diuretics	K	296 (1.5)	1.38 (1.00-1.90)[†]
	Alpha1 blockers (Women)	B	139 (0.7)	1.02 (0.59-1.76)
	Estrogens (women)	B	2 (0.0)	0
GI Ulcer	Nonselective NSAIDs	B/K	897 (4.4)	1.15 (0.92-1.43)
Heart failure	Continued use of NSAIDs (including over-the-counter drugs)	B/K	543 (2.7)	1.01 (0.77-1.34)
	Cilostazol	B	157 (0.8)	1.22 (0.80-1.89)
	Diltiazem, Verapamil	B	129 (0.6)	0.97 (0.58-1.63)
	Lobeglitazone, Pioglitazone	B	35 (0.2)	0
	Dronedarone	B	0 (0.0)	0
Syncope, orthostatic hypotension	Acetylcholine esterase inhibitor	B/K	333 (1.6)	1.15 (0.83-1.60)
	Alpha-1 blockers	B/K	21 (0.1)	0
	Tertiary TCAs	B	23 (0.1)	0

	Chlorpromazine, Thioridazine, Olanzapine	B	3 (0.0)	0
Parkinson's disease	Antipsychotics other than clozapine and quetiapine	B/K	308 (1.5)	0.88 (0.60-1.31)
	Metoclopramide	B	27 (0.1)	0.74 (0.19-2.30)
	Metoclopramide and clebopride	K	33 (0.2)	0.95 (0.31-2.96)
COPD/Asthma	Nonselective beta-blockers	K	617 (3.0)	1.23 (0.98-1.55)
	Oral steroid use instead of inhaled steroid	K	162 (0.8)	0.99 (0.62-1.60)
Type 2 diabetes	Nonselective beta-blockers (exception, sotalol)	K	862 (4.3)	1.20 (0.98-1.47)
	Oral steroids	K	289 (1.4)	1.04 (0.74-1.47)
	Metformin in patients with end-stage renal failure or dialysis	K	79 (0.4)	1.47 (0.85-2.54)
Hypertension	Continued use of NSAIDs (including over-the-counter drugs) in patients using three or more antihypertensive drugs	K	429 (2.1)	1.15 (0.86-1.54)
Renal Failure	NSAIDs	B/K	71 (0.4)	1.14 (0.59-2.20)
	Bisphosphonate	K	5 (0.0)	0
Ischemic Heart Disease	COX2-selective inhibitors	K	211 (1.0)	1.19 (0.80-1.77)
Edema	Continuous use of loop diuretics for simple edema in patients without heart failure, liver cirrhosis, chronic renal failure, or nephrotic syndrome	K	140 (0.7)	1.41 (0.90-2.22)
Insomnia	Oral, nasal decongestants (e.g. pseudoephedrine, phenylephrine)	K	106 (0.5)	1.26 (0.74-2.14)
Esophageal disease	Oral bisphosphonates	K	63 (0.3)	1.51 (0.78-2.92)
Closed angle glaucoma	Strong anticholinergic agents	K	9 (0.0)	3.27 (0.82-13.02)
**DDI-PIM**				
**Object Drug**	**Interacting Drug**			

Any combination of three or more of these CNS-active drugs		B	3,302 (16.3)	1.00 (0.88-1.13)
Multiple anticholinergics		B/K	1,559 (7.7)	1.04 (0.88-1.23)
NSAIDs/Coxib	ACE inhibitor/ARB	K	1,204 (5.9)	1.18 (0.99-1.42)
NSAIDs	Anticoagulants	K	1,037 (5.1)	1.04 (0.85-1.27)
Diuretics	NSAIDs	K	823 (4.1)	1.18 (0.95-1.46)
SSRIs	NSAIDs	K	367 (1.8)	1.09 (0.79-1.52)
Potassium-sparing diuretics	ACE inhibitors /ARB	B	312 (1.5)	0.70 (0.65-1.34)
Multiple ACE inhibitors	ARB	B/K	272 (1.3)	1.19 (0.84-1.69)
Loop diuretics	Peripheral alpha-1 blockers	B	228 (1.1)	1.07 (0.73-1.55)
Oral anticoagulants	Antiplatelet drugs	K	194 (1.0)	1.75 (1.25-2.44) *
Opioids	Benzodiazepines	B/K	161 (0.8)	1.23 (0.80-1.89)
NSAIDs	Oral steroids	B/K	148 (0.7)	1.01 (0.60-1.70)
Opioids	Gabapentin, Pregabalin	B	46 (0.2)	1.34 (0.64-2.82)
Warfarin	NSAIDs	B	37 (0.2)	1.93 (0.92-4.06)
Theophylline	Cimetidine	B	21 (0.1)	1.27 (0.41-4.00)
Warfarin	Macrolides (excluding azithromycin) Quinolones	K	8 (0.0)	3.24 (1.04-10.12)†
Warfarin	Amiodarone	B	8 (0.0)	3.10 (0.77-12.42)
Warfarin	Ciprofloxacin	B	3 (0.0)	2.38 (0.33-17.02)
Theophylline	Ciprofloxacin	B/K	2 (0.0)	6.72 (0.94-47.79)
Loop diuretics	Lithium	B	2 (0.0)	1.27 (0.41-4.00)
Warfarin	Trimethoprim-sulfamethoxazole	B/K	1 (0.0)	0
ACEIs	Lithium	B	1 (0.0)	0
Warfarin	Macrolides (excluding azithromycin)	B	0 (0.0)	0
Phenytoin	Trimethoprim-sulfamethoxazole	B/K	0 (0.0)	0
**Omission-PIM**				
**Disease or Syndrome or Drugs**	**Omission Drugs**			
Experiences a fall, or is at high risk of osteoporosis	Vit D and calcium supplements	K	8,528 (42.0)	1.13 (1.00-1.28)†
Past history of myocardial infarction, coronary stenting, coronary artery bypass surgery, cerebrovascular stent, past stroke, transient	Statin treatment	K	6,747 (33.2)	1.00 (0.91-1.11)

ischemic attack, and peripheral arterial disease				
COPD	Long-acting bronchodilator inhalers	K	5,589 (27.5)	1.07 (0.59-1.95)
Osteoporosis	Osteoporosis treatment	K	5,087 (25.1)	1.04 (0.93-1.16)
Constipation-causing drug	laxatives	K	4,580 (22.6)	1.07 (0.96-1.19)
Past myocardial infarction, coronary stenting, coronary artery bypass surgery, cerebrovascular stent, past stroke, transient ischemic attack, and peripheral arterial vascular disease	Antiplatelet agents	K	4,184 (20.6)	1.10 (0.98-1.22)
Regular opioid analgesics to relieve cancer pain	Prophylactic laxatives	K	977 (4.8)	1.10 (0.90-1.34)
Chronic atrial fibrillation	Oral anticoagulants	K	925 (4.6)	1.10 (0.90-1.33)
Antiplatelet drugs and NSAIDs	PPI	K	844 (4.2)	0.93 (0.74-1.18)
Severe renal impairment (CrCl < 30 mL/min)	Vit D	K	588 (2.9)	1.27 (1.01-1.59)†
Oral steroids	Calcium/ Vit D supplementation and, if necessary, bisphosphonate	K	475 (2.3)	1.05 (0.79-1.39)
Bisphosphonate and denosumab	Calcium/Vit D supplementation	K	121 (0.6)	1.04 (0.57-1.87)
Long-acting opioid analgesics	Short-acting opioid analgesics	K	33 (0.2)	1.53 (0.72-3.26)
Rheumatoid arthritis	Folic acid	K	3 (0.0)	0

PIM, Potentially Inappropriate Medication; ED, Emergency Department; aHR, adjusted Hazard Ratio; Ds-PIM, PIM under specific diseases or conditions; DDI-PIM, PIM due to drug interactions; NSAIDs, Non-Steroidal Anti-Inflammatory Drugs; BPH, Benign Prostatic Hyperplasia; GI, Gastrointestinal Diseases; SSRI, Selective Serotonin Reuptake Inhibitors; ACEI, Angiotensin Converting Enzyme Inhibitors; ARB, Angiotensin Receptor Blockers; COPD, Chronic Obstructive Pulmonary Disease; PPI, Proton Pump Inhibitors; CCB, Calcium Channel Blockers; Vit D, Vitamin D

Adjusted Variables were age, sex, Long-Term Care Grade, Charlson Comorbidity Index, disease (dementia, hypertension, diabetes, Chronic Obstructive Pulmonary Disease, Peptic Ulcer Disease, history of fractures, Ischemic Heart Disease, heart failure, asthma, history of pneumonia, cancer)

*$p < 0.01$, †$p < 0.05$

‡B, Beers Criteria; K, Korean Criteria;
